# Effects of Coenzyme Q_10_ Supplementation on Oxidative Stress Markers, Inflammatory Markers, Lymphocyte Subpopulations, and Clinical Status in Dogs with Myxomatous Mitral Valve Disease

**DOI:** 10.3390/antiox11081427

**Published:** 2022-07-22

**Authors:** Natalia Druzhaeva, Alenka Nemec Svete, Gabrijela Tavčar-Kalcher, Janja Babič, Alojz Ihan, Katka Pohar, Uroš Krapež, Aleksandra Domanjko Petrič

**Affiliations:** 1 Small Animal Clinic, Veterinary Faculty, University of Ljubljana, 1000 Ljubljana, Slovenia; nataliadruzhaeva@gmail.com (N.D.); alenka.nemecsvete@vf.uni-lj.si (A.N.S.); 2 Institute of Food Safety, Feed and Environment, University of Ljubljana, 1000 Ljubljana, Slovenia; gabrijela.tavcarkalcher@vf.uni-lj.si (G.T.-K.); janja.babic@vf.uni-lj.si (J.B.); 3 Institute of Microbiology and Immunology, Faculty of Medicine, University of Ljubljana, 1000 Ljubljana, Slovenia; alojz.ihan@mf.uni-lj.si (A.I.); katka.pohar@mf.uni-lj.si (K.P.); 4 Institute of Poultry, Birds, Small Mammals and Reptiles, Veterinary Faculty, University of Ljubljana, 1000 Ljubljana, Slovenia; uros.krapez@vf.uni-lj.si

**Keywords:** coenzyme Q_10_, ubiquinone, supplementation, myxomatous mitral valve disease, congestive heart failure, dog, oxidative stress, inflammation, lymphocytes, neutrophils

## Abstract

Scarce data exist on the effects of coenzyme Q_10_ (CoQ_10_) supplementation in dogs with myxomatous mitral valve disease (MMVD). The purpose of this study was to investigate the effect of CoQ_10_ supplementation on oxidative stress markers (glutathione peroxidase, F2-isoprostanes), markers of inflammation (tumor necrosis factor-α, TNF soluble receptor II, leucocytes, and their subtypes), lymphocyte subpopulations (T helper and cytotoxic T lymphocytes, including activated T lymphocytes, and B lymphocytes), and echocardiographic and clinical parameters in dogs with MMVD. In this randomized, controlled, double-blind, longitudinal study, 43 MMVD dogs in stages ACVIM (American College of Veterinary Internal Medicine classification) B2 and ACVIM C and D (congestive heart failure (CHF)) received water-soluble coenzyme Q_10_ (100 mg twice daily) or placebo for 3 months, and 12 non-supplemented healthy dogs served as controls. All parameters were measured before and after supplementation in MMVD dogs and once in healthy dogs. CoQ_10_ supplementation had a positive impact on neutrophil percentage, lymphocyte percentage, and lymphocyte concentration in our cohort of dogs with CHF (ACVIM C and D). Conclusion: CoQ_10_ as an oral supplement may have benefits in terms of decreasing inflammation in dogs with MMVD and CHF.

## 1. Introduction

Coenzyme Q_10_ (CoQ_10_), or ubiquinone, is an endogenously synthesized lipid-soluble compound which is essential for the functioning of all cells of an animal’s body. It is present in the membranes of mitochondria and other cell organelles, cell membranes, cytoplasm, and blood plasma. Coenzyme Q_10_ plays a major role in ATP production and antioxidant defense, and it directly or indirectly regulates many bodily functions, including the functioning of the immune system [1,2,3].

Oxidative stress, defined as “an imbalance between oxidants and antioxidants in favor of the oxidants, leading to a disruption of redox signaling and control and/or molecular damage” [4], is present in human cardiovascular diseases (CVD) and heart failure (HF) [5,6]. Low levels of plasma and myocardial CoQ_10_ have been found in human CVD [7,8,9,10]. Due to the known fact of CoQ_10_ deficiency in people with CVD, the role of CoQ_10_ in energy production, and its antioxidant potential, supplemental CoQ_10_ has long been studied as an adjunctive treatment in human HF patients [11,12,13,14,15,16].

Myxomatous mitral valve disease (MMVD) is a degenerative disease of the mitral and/or tricuspid valve and is the most common cardiac pathology in dogs [17]. As such, it is also the most common cause of canine congestive heart failure (CHF), a debilitating condition which shortens the lifespan of an animal and lowers the quality of life of both the dog and its owner. Treatment of MMVD and CHF is mostly conservative, with surgical mitral valve repair only rarely performed in dogs due to the high cost and relative inaccessibility [17]. The search for new medical agents and dietary supplements with the potential to slow the progression of the disease is ongoing. In accordance with studies, oxidative stress is present in canine cardiac diseases, including MMVD [18,19,20]. Myocardial CoQ_10_ deficiency has recently been confirmed in Cavalier King Charles Spaniels with CHF due to MMVD [21], and although plasma CoQ_10_ deficiency has not been confirmed in dogs with MMVD or other cardiac pathologies [22,23], CoQ_10_ has drawn interest as a dietary supplement which could potentially positively impact the course of MMVD in dogs due to its antioxidant and other properties. In contrast to human medicine, few CoQ_10_ supplementation studies have been conducted in canine cardiac patients with spontaneous disease [24,25], and no studies have assessed the effect of supplemental CoQ_10_ on oxidative stress parameters.

Inflammation, which is the other hallmark of human [26] and canine [27,28,29,30] heart failure (HF), has been shown to be linked to oxidative stress in both people [31,32] and dogs [20,30] with cardiac diseases. The reducing effect of CoQ_10_ supplementation on inflammatory markers has been found in coronary artery disease (CAD) and other chronic inflammatory diseases in people [3,33,34,35]. It is hypothesized that CoQ_10_ exerts an anti-inflammatory effect by decreasing reactive oxygen species (ROS) concentrations and subsequently reducing nuclear factor kappa B (normally activated by ROS) gene expression [35]. The effect of CoQ_10_ on markers of inflammation has not been studied in dogs with heart diseases.

Besides markers of inflammation, lymphocyte subpopulations have also been reported to be altered in both people with HF [36,37] and dogs with cardiac diseases, including MMVD and CHF [38,39]. In a study of dogs with MMVD conducted by our group [39], a lower percentage of T helper lymphocytes, a higher percentage of cytotoxic T lymphocytes, and a lower T helper/cytotoxic T lymphocyte ratio were documented in dogs with CHF due to MMVD compared to healthy controls, whereas these alterations were not present in the non-CHF group of participating MMVD patients. As far as we know, the effect of CoQ_10_ supplementation on lymphocyte subtypes has not been studied in dogs with heart diseases.

To date, three studies have been conducted to assess the effects of CoQ_10_ supplementation in canine cardiac patients. A few parameters were tested, and some possible benefits were detected in two of these studies [24,25,40]. An earlier study in experimental tachycardia-induced CHF showed that dogs with CHF supplemented with CoQ_10_ for six weeks in total had lower filling pressures in an early stage of CHF and less hypertrophy in severe CHF when compared to non-treated dogs [40]. In the other study, the authors reported, over a period of 28 days of supplementation, an increased shortening fraction and M-mode-derived ejection fraction in a group of dogs of less than 6 kg, which could be just a consequence of a larger end-diastolic diameter [24]. The latest (single-blind, crossover) study [25] to investigate the potential benefits of CoQ_10_ in canine MMVD did not show any positive effects on the severity of the disease and owner-perceived quality of life in Cavalier King Charles Spaniels supplemented with CoQ_10_ for three weeks. The authors of the mentioned study concluded that a long-term placebo-controlled trial is warranted in dogs with MMVD to determine the long-term efficacy of CoQ_10_ on the clinical severity of MMVD.

The aim of the present study was to assess the effect of oral CoQ_10_ supplementation given for three months in addition to standard cardiac therapy on oxidative stress parameters (glutathione peroxidase (GPX), F2-isoprostanes), inflammatory parameters (tumor necrosis factor-α (TNF-α), soluble TNF receptor II (TNFSR-II), leucocyte populations), lymphocyte subpopulations, cardiac biomarkers (N-terminal pro B-type natriuretic peptide (NT-proBNP) and cardiac troponin I (cTnI)), selected echocardiographic parameters, and clinical signs in dogs of different breeds with spontaneous MMVD with and without CHF.

## 2. Materials and Methods

### 2.1. Animals

Dogs with MMVD in stages ACVIM (American College of Veterinary Internal Medicine classification) B2 and ACVIM C and D, as well as healthy control dogs, were recruited for the study. The inclusion process took place between 4 December 2018 and 28 December 2020 at the Small Animal Clinic of the University of Ljubljana. Clinical examination, echocardiography, electrocardiography (if indicated), thoracic radiography (if indicated), routine hematology, and biochemistry analyses were performed at inclusion. All diagnostic procedures were performed by an experienced veterinarian. Diagnosis of MMVD was in all cases confirmed by echocardiography and that of CHF by thoracic radiography and echocardiography. All healthy dogs were subjected to the same diagnostic tests to confirm their eligibility for the healthy control group.

#### 2.1.1. Inclusion Criteria

Dogs were classified as ACVIM B2 if they were asymptomatic (without a history of heart failure) and had advanced mitral regurgitation with subsequent left-sided cardiac remodeling. The criteria for inclusion in this group were as follows: Grade 3/6 cardiac murmur intensity, echocardiographic LA/Ao (left atrial/aortic ratio) ≥ 1.6 in early diastole, left ventricular internal end-diastolic diameter normalized to body weight (nLVIDd)^1/3^ ≥ 1.7 [17] measured in the right short-axis view, and no evidence of comorbidities at the time of clinical examination and in routine hematology and biochemistry findings. Dogs were included in the cardiac failure group (ACVIM C and D) if they were symptomatic (current or previous symptoms of cardiac failure), had echocardiographically confirmed MMVD, had current or previously diagnosed cardiogenic pulmonary edema on chest radiographs, and had no evidence of comorbidities at the time of clinical examination and in routine hematology and biochemistry findings. Healthy control dogs were included in the control group if they were clinically healthy, had no evidence of cardiac disease on echocardiographic examination, and routine hematology and biochemistry findings showed no evidence of disease.

#### 2.1.2. Exclusion Criteria

Dogs with concomitant diseases (including chronic kidney disease and other metabolic diseases, systemic or local inflammation, and neoplasia) were excluded. Dogs that had not received complete treatment for cardiac disease (pimobendan for B2 dogs or heart failure treatment for ACVIM C and D dogs) at least four weeks before inclusion were not included until they met these criteria. Critically ill dogs were not included until their condition became stable with recommended treatment [17]. Dogs receiving (or having received in the past month) glucocorticoids or other immunosuppressive agents, antibiotics, or dietary supplements were excluded.

### 2.2. Study Design

In this randomized, double-blinded, placebo-controlled study, we included dogs with MMVD receiving water-soluble CoQ_10_ in a daily dose of 200 mg (100 mg twice a day) or an organoleptically matched placebo for three months. The daily dose of 200 mg was chosen based on the results of our previous research [23]. Dogs were included in the study after complete diagnostic procedures and allocated to either the ACVIM B2 (non-CHF) group or the ACVIM C and D (CHF) group and then further blindly randomized to receive CoQ_10_ or placebo. Randomization was performed by one of the authors (A.N.S.) not involved in the assessment, diagnostics, treatment, following of patients, or any communication with the owners. All diagnostic procedures (including clinical examination, echocardiography, and blood tests) were performed twice in MMVD dogs, i.e., on the day of inclusion just before the start of supplementation and at the end of the study, approximately 12 h after the final dose of the supplement. Diagnostic procedures, treatment, and all communication with the owners were performed by A.D.P. and N.D., both blinded to the type of supplement the dogs were receiving during the study. At the time of inclusion, all owners completed a questionnaire regarding the dog’s diet and the supplements and medications the dogs were receiving. An additional questionnaire regarding the dog’s current symptoms was completed at the beginning and at the end of the study. Owners were instructed on the correct administration and the storage of the supplement.

Water-soluble CoQ_10_ (ubiquinone; Q10Vital liquid, Valens, Šenčur, Slovenia) in the form of a 7.5% water suspension (derived from CoQ_10_, Ubidecarenone (Xiamen Kingdomway Group Co, Xiamen, China) in an inclusion complex with β-cyclodextrin [41]) was used in the study. A single dose comprising 100 mg of CoQ_10_ was equivalent to 1.333 g of the suspension, with the daily dose being equal to 2.667 g. The matched placebo was comprised of cyclodextrin, water, food colorants, and the preservative methyl 4-hydroxybenzoate sodium salt. Both CoQ_10_ suspension and the placebo were packed in identical plastic bottles and put in opaque bags by a person not involved in any diagnostics or treatment before being handed out to veterinarians and subsequently to dog owners. All those performing any status assessment or analyses were blinded to the type of supplement the dogs were receiving.

Healthy dogs underwent clinical and echocardiographic examination only once. They did not receive dietary supplements according to the study protocol. A questionnaire regarding the dog’s diet, any treatments, and dietary supplements was completed before enrollment in the study.

### 2.3. Blood Sampling

Blood was taken by jugular or cephalic venipuncture after 12 h of fasting. The last dose of supplement (CoQ_10_ or placebo) at the end of the supplementation period was given 12 h prior to sampling. Samples were drawn into EDTA-containing tubes (for complete blood count (CBC) with white blood cell (WBC) differential count, flow cytometry, NT-proBNP, F2-isoprostanes (8-iso-prostaglandin F2α (8-isoPGF2α))), serum separator tubes (for routine biochemistry, cTnI, CRP, TNF-α, TNFSR-II), and lithium-heparin tubes (for CoQ_10_, whole blood GPX activity). Samples for routine hematology and biochemistry were analyzed within 1 h after sampling. Samples for flow cytometry were stored in darkness at room temperature and were analyzed within 24 h. Samples for NT-proBNP, F2-isoprostanes, and CoQ_10_ were immediately centrifuged at 1500 × g at 4 °C for 15 min, and separated plasma was stored at −80 °C until analysis. Samples for cTnI, CRP, TNF-α, and TNFSR-II were centrifuged after complete coagulation at 1300 × g at room temperature for 10 min, and serum was stored at −80 °C until analysis.

#### 2.3.1. Routine Hematology and Biochemistry Analyses

Hematologic analysis was performed with an automated laser-based hematology analyzer (ADVIA 120, Siemens, Munich, Germany). Biochemistry analysis (glucose, urea, creatinine, alanine aminotransferase, alkaline phosphatase, total protein, albumin) was performed with an automated biochemistry analyzer (RX Daytona, Randox, Crumlin, UK). Electrolytes (sodium, potassium, chloride) were measured with an electrolyte analyzer (ILyte, Instrumentation laboratory, Lexington, Massachusetts).

#### 2.3.2. Determination of Oxidative Stress Markers

CoQ_10_ was measured on a liquid chromatography-tandem mass spectrometry system (Acquity UPLC with Xevo TQ detector, Waters, Milford, MA, USA), as described elsewhere [23]. Whole blood GPX activity was measured spectrophotometrically with an automated biochemistry analyzer (RX Daytona, Randox, Crumlin, UK) using a commercial Ransel reagent kit (Ransel, Randox, Crumlin, UK), which is based on the method of Paglia and Valentine [42]. Concentrations of plasma F2-isoprostanes were measured with a commercially available canine-specific ELISA kit (Canine 8-iso-Prostaglandin F2a (8-isoPGF2α) ELISA kit; MyBioSource.com). The assays were performed according to the original manufacturer’s instructions, with all samples assayed in duplicate.

#### 2.3.3. Determination of Inflammatory Markers

Concentrations of serum CRP, TNF-α, and TNFSR-II were measured with commercially available canine-specific ELISA kits (Canine CRP ELISA; Alpco, Salem, NH, USA; Canine TNF-alpha Quantikine ELISA kit; R&D Systems, Minneapolis, MN, USA; Canine Tumor Necrosis Factor Soluble Receptor II (TNFSR-II) ELISA kit; MyBioSource.com). Assays were performed according to the original manufacturer’s instructions, with all samples assayed in duplicate.

#### 2.3.4. Flow Cytometry

Flow cytometric analysis of whole blood samples to determine the percentage of lymphocyte subpopulations, including T lymphocytes (CD3+), T helper cells (CD3+CD4+), activated T helper cells (CD3+CD4+CD25+), cytotoxic T lymphocytes (CD3+CD8+), activated cytotoxic T lymphocytes (CD3+CD8+CD25+), double-positive T lymphocytes (DPT) (CD3+CD4+CD8+), double-negative T lymphocytes (DNT) (CD3+CD4-CD8-), and B lymphocytes (CD45+CD21+), was performed as described previously [39]. Briefly, the whole-blood lysis method was used according to the manufacturer’s protocol [43]. Blood samples were incubated with monoclonal antibodies at 2 to 8 °C for 30 min. After incubation, the erythrocytes were lysed with a red blood cell lysis solution. The cells were washed with 0.1% bovine serum albumin in phosphate-buffered saline, resuspended in 0.1% bovine serum albumin in phosphate-buffered saline, and analyzed using a FACSCanto II flow cytometer (BD Biosciences, San Jose, California). The flow cytometer was calibrated using BD FACSDiva CS &T Research Beads. Compensation controls were performed to correct for fluorescence spillover. Absolute concentrations of PBL subtypes were calculated based on differential counts of CBC and WBC and flow cytometry results.

#### 2.3.5. Determination of Cardiac Biomarkers

For measurement of plasma NT-proBNP concentrations, IDEXX ELISA (IDEXX Laboratories, Leipzig, Germany) was used. For measurement of serum cTnI concentrations, a high-sensitivity immune-assay (ADVIA Centaur TnI-Ultra; Siemens) was used.

### 2.4. Echocardiographic and Clinical Assessment

Echocardiography (2-D, M-mode, color, and spectral Doppler (GE Vivid E9, General Electric Healthcare)) was performed before and after the supplementation period. Standardized transthoracic views were used [44]. Dogs were classified as ACVIM B2 (non-CHF) when asymptomatic with cardiac remodeling of the left ventricle and left atrium due to MMVD or ACVIM C and D (CHF due to MMVD), based on the echocardiographic measurements, signs of pulmonary edema on thoracic radiographs, and clinical signs [17]. Due to the large number of measured parameters in this study, only selected echocardiographic measurements were included in the study (LA/Ao, nLVIDd, normalized left ventricular internal systolic diameter (nLVIDs), mitral valve E-wave (MV E) velocity, mitral valve A-wave (MV A) velocity, MV E/A ratio, tricuspid regurgitation pressure gradient (TR PG), and fractional shortening (FS)).

### 2.5. Statistical Analysis

The data were analyzed using IBM SPSS (version 25, IBM Corp., Armonk, NY, USA). Descriptive statistics were used to obtain basic information about the variables measured. Due to the small number of dogs included in the individual groups, non-parametric tests were used to assess differences in measured parameters. Comparisons of measured parameters were performed within supplemented groups before and after a 3-month supplementation period with placebo or CoQ_10_ in ACVIM B2 and CHF patients (Wilcoxon signed-rank test), and between supplemented groups—between the placebo and the CoQ_10_ groups before and after a 3-month supplementation period in ACVIM B2 and CHF patient groups (Mann–Whitney test). Furthermore, in each patient group (ACVIM B2 and CHF) supplemented either with placebo or CoQ_10_, the change in a measured parameter was calculated (delta: the value after supplementation minus the value before supplementation) and the changes (deltas) between placebo and CoQ_10_ groups of ACVIM B2 and CHF patients were compared using the Mann–Whitney test. Additionally, all measured parameters were compared between groups of patients (ACVIM B2 and CHF) and healthy dogs (control group) before supplementation using the Kruskal–Wallis test, followed by pairwise comparisons and Bonferroni adjustments. The results were expressed as medians and interquartile ranges (IQR; 25th to 75th percentiles). A value of *p* < 0.05 was considered significant.

## 3. Results

**Figure 1:**
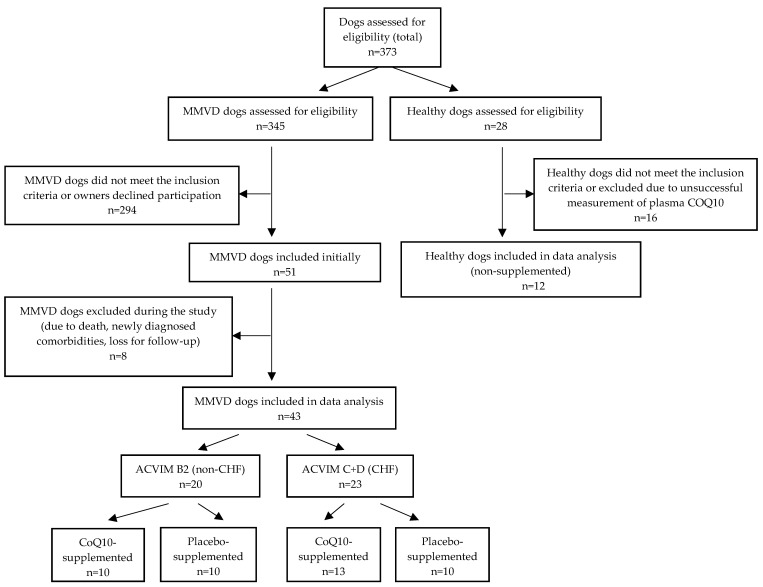
The flow diagram of dogs with MMVD and healthy dogs during the study.

Fifty-five client-owned dogs of different breeds (43 dogs with MMVD and 12 healthy control dogs) were included in the study. The inclusion process is shown in Figure 1. The baseline characteristics of all included dogs are shown in Table 1.

### 3.1. Within-Group Comparisons of Measured Parameters over a Three-Month Supplementation Period

The results of the within-group comparisons of the measured parameters are shown in Table 2 and Table 3 for the ACVIM B2 group and the CHF group, respectively. In both patient groups, three months of CoQ_10_ supplementation resulted in a significant increase in plasma CoQ_10_ concentration compared with baseline. During three months of supplementation, none of the other measured parameters changed significantly in the CoQ_10_- or placebo-supplemented groups of ACVIM B2 patients (Table 2), whereas in the CHF group (Table 3), supplementation with CoQ_10_ resulted in a significant change in DNT and FS.

The concentration of TNF-α was under the detection limit and as such was not included in the statistical analyses.

### 3.2. Comparisons of Measured Parameters between the CoQ_10_ and Placebo Groups before and after Three-Month Supplementation

The results of the comparisons of measured parameters between the CoQ_10_-supplemented group and placebo-supplemented group are shown in Table 2 (for ACVIM B2) and Table 3 (for ACVIM C and D). In ACVIM B2 patients, baseline concentrations of measured parameters did not differ between the CoQ_10_-supplemented and placebo-supplemented dogs, except for nLVIDd, whereas in the CHF group, none of the baseline concentrations of measured parameters differed significantly between the CoQ_10_-supplemented and placebo-supplemented groups.

After the three-month supplementation period, plasma CoQ_10_ concentrations were significantly higher in the CoQ_10_-supplemented group than in the placebo-supplemented group in ACVIM B2 (Table 2) and CHF dogs (Table 3). No other parameters differed significantly in either patient group.

### 3.3. Comparisons of Changes (Deltas) in Measured Parameters between CoQ_10_-Supplemented and Placebo-Supplemented Groups

In both patient groups, ACVIM B2 and CHF, a significantly higher change (increase) in plasma CoQ_10_ concentration was observed in the CoQ_10_-supplemented groups compared with the change in this parameter in the corresponding placebo groups (Table 2 and Table 3). In ACVIM B2 patients (Table 2), no significant differences in the change in other parameters were observed between the placebo and CoQ_10_-supplemented groups, whereas in CHF patients (Table 3), a significant difference in the change (increase or decrease) in several parameters was observed between the placebo and CoQ_10_-supplemented groups.

### 3.4. Comparisons of Measured Parameters between ACVIM B2, CHF, and Healthy Groups

The results of the comparison of measured parameters between ACVIM B2, CHF, and healthy dogs are shown in Table 1 and Table 4. Healthy dogs were significantly younger than those in the ACVIM B2 and CHF groups (Table 1). In addition, statistical analysis revealed significant differences in numerous parameters between dog groups, as indicated in Table 4.

### 3.5. Owner-Perceived Assessment of the Condition of a Dog at the end of the Supplementation Period Compared to That at the Beginning (Subjective Assessment, Not Included in the Statistics)

Out of ten owners of ACVIM B2 dogs supplemented with CoQ_10_ (10 dogs), four assessed the overall condition of the dog as better, four similar, and two worse than it was before the start of supplementation. For ACVIM B2 placebo-supplemented dogs (ten dogs), the results of the overall condition of the dog were as follows: three better, seven similar, and none worse than that prior to supplementation. For CHF CoQ_10_-supplemented dogs (thirteen dogs), the results of the overall condition of the dog were as follows: seven better, four similar, and two worse than that prior to supplementation. For CHF placebo-supplemented dogs (ten dogs), the results of the overall condition of the dog were as follows: two better, two similar, and six worse than that prior to supplementation.

### 3.6. Adverse Effects

No significant adverse effects were noticed during the study. Short-lived diarrhea which did not require treatment was noted in 3 out of 23 dogs receiving CoQ_10_ and in 4 out of 20 dogs receiving placebo during the study period.

## 4. Discussion

In the present study, we could not confirm the positive effects of CoQ_10_ supplementation on oxidative stress markers, lymphocyte subpopulations, markers of disease severity (circulating cardiac biomarkers and echocardiographic parameters), and TNFSR-II as a marker of inflammation; however, a positive effect was noted on selected inflammatory parameters (neutrophil percentage and lymphocyte percentage and concentration) in dogs with CHF. A daily dose of 200 mg of CoQ_10_ (given as 100 mg twice per day) was well-tolerated and significantly increased plasma CoQ_10_ concentration in both CoQ_10_-supplemented groups (ACVIM B2 and CHF) in comparison to their basal concentrations and to the concentration of plasma CoQ_10_ measured in corresponding placebo groups.

Out of all significant differences detected during data analysis (Table 2 and Table 3), only those found when comparing CoQ_10_-supplemented groups to placebo-supplemented groups could be contributed to CoQ_10_ supplementation. The most important of them were the significant differences in the median change (delta) of neutrophil percentage and lymphocyte percentage and concentration between the CoQ_10_-supplemented and placebo-supplemented groups of dogs with CHF. Our results showed that CHF dogs who received CoQ_10_ had a net decrease in their neutrophil percentage during the study period (negative delta), while dogs who received a placebo had a net increase in this parameter (positive delta). CoQ_10_-supplemented dogs had a net increase in lymphocyte count and percentage (positive delta), while placebo-supplemented dogs had a net decrease in these parameters (negative delta), with all mentioned differences being significant.

An increase in neutrophil count and decrease in lymphocyte count are typical for systemic inflammation, and it is known that low-grade inflammation is present in CHF in people [45,46] and dogs [29,30]. A higher neutrophil percentage has been found in dogs with advanced-stage CHF in comparison to dogs with stable CHF and/or non-CHF dogs or healthy controls [29,30,47]. Low lymphocyte counts are often found in human CVD and were shown to be a predictor of mortality in CVD patients [48,49,50], but the results of studies in dogs are contradictory, with most of them not detecting decreased lymphocyte count or percentage in canine patients with heart disease [29,30,38,39,47]; however, in one of the mentioned studies, a significantly lower lymphocyte percentage was found in CHF and non-CHF groups of canine cardiac patients compared to that in healthy dogs [30]. In another study, a lower lymphocyte count was shown in dogs with advanced-stage CHF in comparison to that in dogs with stable CHF and healthy controls [38]. In the current study, our CHF group’s neutrophil percentage (as well as lymphocyte percentage and concentration) did not differ significantly from ACVIM B2 or healthy dogs at baseline (Table 4). This is likely related to the very selective inclusion process. Pronounced inflammation is most anticipated in untreated, unstable, or critically ill patients [29]; however, only dogs who were receiving proper treatment and were not critically ill were included in our study.

Despite the absence of significant differences in neutrophils and lymphocytes between cardiac patients in CHF and healthy dogs at the baseline (Table 4), during the three-month supplementation period, neutrophil percentage rose in dogs receiving placebo and fell in those receiving CoQ_10_, and lymphocyte percentage and concentration fell in placebo-supplemented patients and rose in those receiving CoQ_10_. The positive effect of CoQ_10_ supplementation may be the result of CoQ_10_’s anti-inflammatory properties. The anti-inflammatory effect of CoQ_10_ has been previously studied in people [3,51]. The association between oxidative stress and inflammation has been shown in dogs with cardiovascular diseases [30], but the anti-inflammatory effect of CoQ_10_ supplementation has not been studied in canine patients. Our study is the first to report the possible benefits of oral CoQ_10_ supplementation in combating inflammation in dogs with CHF. However, in our study, CoQ_10_ did not affect TNFSR-II (despite that this parameter was significantly higher in CHF dogs compared to that in ACVIM B2 and healthy dogs at baseline (Table 4)). No previous studies have assessed the effect of oral CoQ_10_ on serum TNFSR-II concentration. Instead, the effect on serum TNF-α level was examined. These studies showed that CoQ_10_ supplementation decreased the level of TNF-α in a wide range of diseases, including CVD [51,52,53]. In our study, we could not assess this as in all dogs but one, TNF-α concentration was under the limit of detection.

Lymphocyte subpopulations were not affected by CoQ_10_ supplementation in our study. The differences in lymphocyte subtypes previously described in dogs with CHF in comparison to healthy dogs [38,39] were also not observed in the present cohort of dogs, possibly because most dogs included in this study were in stable CHF and none were critically ill, whereas many critically ill patients were included in our previous cross-section study [39]. However, we found a significantly lower T helper percentage and CD4/CD8 ratio and a significantly higher percentage of cytotoxic T lymphocytes in ACVIM B2 patients compared with CHF patients and healthy dogs, which we believe may be because the group included the oldest participants or due to advanced cardiac remodeling in this group. However, the dogs in the ACVIM B2 group were significantly older than the healthy dogs but not than those with CHF.

In our study, CoQ_10_ as a supplement did not affect selected oxidative stress markers (plasma F2-isoprostanes concentration and whole blood GPX activity). Being an established lipid peroxidation marker [54,55], F2-isoprostanes were found to be increased in the plasma of human HF patients and are linked both to antioxidant status and heart disease severity [56]. Additionally, urinary 15-F2t-isoprostane concentration was found to be increased in advanced CHF and correlated to the disease severity in non-ischemic CHF in people [57]. In dogs with cardiac diseases, serum 8-F(2alpha)-isoprostanes were found to be significantly higher in CHF in comparison to a healthy control group [18]. In our study, plasma F2-isoprostane concentration did not differ significantly between both groups of MMVD dogs and healthy controls at baseline (Table 4), and no effect of CoQ_10_ supplementation on this parameter was noted. Likewise, whole blood GPX activity, which is another marker of oxidative stress, did not differ between cardiac patients and healthy dogs (similar to another study in dogs [58]) and was not affected by CoQ_10_ supplementation. In people, the results of studies researching the effect of supplementary CoQ_10_ on whole blood GPX activity in different diseases including CVD are not consistent either [34,59,60]. In the present study, orally administered CoQ_10_ did not have an effect on the studied parameters of oxidative stress despite a huge increase (1.7-fold to 11.5-fold) in plasma CoQ_10_ concentration in all but one CoQ_10_-supplemented dog and a significant increase in all CoQ_10_-supplemented groups in comparison to their basal plasma CoQ_10_ concentrations and placebo. In future CoQ_10_ supplementation studies in canine cardiac patients, other oxidative stress markers may be chosen for the assessment of possible benefits of supplementation.

No positive effects of CoQ_10_ supplementation on echocardiographic parameters were detected in our study. In our CoQ_10_-supplemented dogs with CHF, nLVIDs increased, with the positive change being significantly different from the negative change in placebo-supplemented patients. The latest study published on the effects of CoQ_10_ supplementation in MMVD dogs did not find any positive effect on echocardiographic parameters, which is in accordance with our results [25].

Cardiac biomarkers (cTni and NT-proBNP) were also not affected by CoQ_10_ supplementation, a result similar to that in previous studies in dogs with MMVD [24,25]. Owner-perceived quality of life with subjective assessment of changes in the health condition of dogs participating in our study was assessed by a questionnaire, with data not included in the statistics. Subjectively, in CoQ_10_-supplemented CHF dogs, there were more owners (out of a total number of participants) who noticed improvements in the condition and mood of their dogs in comparison to owners of dogs supplemented with the placebo. In the only study which assessed the quality of life of dogs supplemented with CoQ_10_, no impact was noted [25]. Since the questionnaire used in the present study was not validated and the data were not statistically analyzed, we cannot draw any conclusions regarding improvement in clinical signs. Results in people have also been inconclusive, with some studies reporting increasing quality of life [61] and others reporting no change [62].

As previously mentioned, for the initial assessment of cardiac patients, the studied parameters were compared between three groups of participating dogs (all ACVIM B2 dogs, all CHF dogs, and healthy dogs) with no regard to the type of later assigned supplement. Data are shown in Table 1 (for age and plasma CoQ_10_ concentration) and Table 4 (for other parameters). Although the main goal of this research was to assess the effects of CoQ_10_ supplementation on selected laboratory and clinical parameters, it is worth mentioning that in both groups of our MMVD patients (ACVIM B2 and CHF), plasma CoQ_10_ concentration was significantly higher in comparison to that in healthy dogs. These results differed from those obtained in our previous research [23], where the basal plasma CoQ_10_ concentration in CHF dogs did not differ significantly from that of healthy dogs (dogs with ACVIM B2 were not included in that study); however, our results are in accordance with another study in which CHF patients receiving cardiac treatment had significantly higher plasma CoQ_10_ concentrations compared to those not receiving cardiac treatment and healthy dogs [22]. To the best of our knowledge, to date, no studies in canine cardiac patients have shown decreased levels of plasma CoQ_10_. This is in contrast to the results of studies in human cardiovascular patients, where plasma CoQ_10_ levels have been found to be lower than those in healthy subjects and associated with disease severity and mortality [8,63,64]. Despite this discrepancy in results for plasma levels of CoQ_10_ between people and dogs, there is recent evidence that myocardial levels of CoQ_10_ are lower in Cavalier King Charles Spaniels with CHF compared to non-CHF (ACVIM B1 and B2) dogs and healthy controls [21]. This might support the need for future CoQ_10_ supplementation studies in canine CHF patients despite normal or increased plasma CoQ_10_ concentrations.

Our study has some limitations. The most important of these is the relatively low number of dogs included. The inclusion criteria were restrictive as we did not recruit critically ill dogs, those receiving nutritional supplements, or those with comorbidities, all of which made up a large proportion of the dogs assessed for potential participation (Figure 1). We also did not include dogs that were not receiving proper treatment, and dogs that began treatment were included only after at least one month of proper therapy. Some of the dogs included died or were euthanized during the study and are therefore not included in the statistical analysis (Figure 1). Another limitation may be the short duration of the supplementation period. Although our three-month study is the longest study of CoQ_10_ supplementation in dogs with heart diseases, a longer study could potentially show more benefits of oral CoQ_10_ supplementation. On the other hand, the results of a longer study might be difficult to assess due to the number of dogs with heart failure worsening during the supplementation period. Another limitation of our study is that the dose of CoQ_10_ was the same for all dogs and was not based on body weight, as suggested by other authors [24]. MMVD is typical in smaller dog breeds, and all dogs in our study were small or medium-sized (with the heaviest weighing 20.9 kg). The results of our previous dose-ranging study showed that 200 mg per day was sufficient to achieve a 3-fold increase in all supplemented dogs with MMVD regardless of their weight [23], and in the current study, this dose was sufficient to achieve at least a 3-fold increase in most dogs (19 out of 23), including 2 of our cardiac patients who were the heaviest, weighing 17 and 20.9 kg. In addition, for nutritional supplements it might prove impractical to base the dose on the exact weight, although we can suggest that for studies of other cardiac diseases typical of larger breeds, dose adjustment may be used. Additionally, in our study, TNF-α concentration was under the detection limit in all samples but one. The impact of CoQ_10_ supplementation on TNF-α may be assessed in future supplementation studies in canine cardiac patients using different methods, e.g., fluorescent bead-based assays (Luminex Xmap technology) instead of the ELISA method.

## 5. Conclusions

Our study is the longest of all published trials evaluating the effects of CoQ_10_ supplementation in dogs with heart diseases. Of all parameters assessed, only neutrophil percentage and lymphocyte percentage and concentration were positively affected by supplementation, which may indicate the anti-inflammatory role of CoQ_10_ in systemic inflammation in dogs with CHF due to MMVD. Studies with a longer supplementation period and a larger number of dogs or studies examining the effect of CoQ_10_ on survival are warranted.

## Figures and Tables

**Table 1:** Baseline characteristics of dogs with MMVD (stages ACVIM B2 and C, D) and healthy dogs.

	ACVIM B2	ACVIM C, D	Healthy
Number	20	23	12
Sex (f/m)	9/11	9/14	6/6
Spayed/neutered	9/4	8/4	5/4
Age (years) Median (IQR)	11.7 * (9.6–13.5) *p* = 0.001	10.7 * (9.3–11.8)*p* = 0.019	7.9 (6.1–9.5)
Weight (kg) Median (IQR)	8.4 (6.8–11.5)	7.8 (6.0–11.4)	7.2 (4.8–13.4)
CoQ_10_ (mg/L) Median (IQR)	0.176 * (0.125–0.213)*p* = 0.019	0.171 * (0.145–0.213) *p* = 0.008	0.095 (0.070–0.143)
Breeds	5 CKCS, 4 MB, 1 PEK, 1 CHI, 1 MSCH, 1 LA, 1 NT, 1 MLT, 1 TS, 1 ECS, 1 SHI, 1 CC, 1 HAV	11 CKCS, 3 SHI, 2 MB, 2 PEK, 1 MSCH, 1 MLT, 1 ACS, 1 CHI, 1 MP	7 MB, 3 SHI, 1 YT, 1 TS
Treatment Pimobendan ACE inhibitor Furosemide or torasemide Spironolactone Theophylline Sildenafil Amlodipine Potassium chloride	20 11 - - 6 1 - -	23 23 23 4 1 - 5 3	- - - - - - - -

* Significant (*p* < 0.05) difference in comparison to healthy dogs. Abbreviations: ACE, angiotensin-converting enzyme; ACS, American Cocker Spaniel; ACVIM, American College of Veterinary Internal Medicine; CC, Chinese Crested Dog; CHI, Chihuahua; CKCS, Cavalier King Charles Spaniel; CoQ_10_, coenzyme Q_10_; ECS, English Cocker Spaniel; f, female; HAV, Havanese Dog; IQR, interquartile range; LA, Lhasa Apso; m, male; MB, mixed breed dog; MLT, Maltese; MMVD, myxomatous mitral valve disease; MP, Miniature Poodle; MSCH, Miniature Schnauzer; NT, Norfolk Terrier; PEK, Pekingese; SHI, Shi Tzu; TS, Tibetan Spaniel; YT, Yorkshire Terrier.

**Table 2:** Effect of CoQ_10_ (or placebo) supplementation on selected parameters in dogs with MMVD, stage ACVIM B2. Medians and interquartile ranges (IQR) are shown.

Parameter	CoQ_10_ Supplemented				Placebo						
	Baseline	After 3 Months	*p* *	Delta	Baseline	After 3 Months	*p* *	Delta	*p* ^a^	*p* ^b^	*p* ^c^
CoQ_10_ (mg/L)	0.190 (0.129–0.271)	1.050 (0.560–1.364)	**0.007**	0.790 (0.439–1.118)	0.158 (0.123–0.199)	0.174 (0.140–0.212)	0.646	−0.009 (−0.024–0.028)	0.364	**<0.001**	**<0.001**
GPX (U/g HGB)	758.0 (692.8–794.8)	765.5 (725.1–793.8)	0.333	11.5 (−13.1–28.5)	701.0 (643.7–806.1)	668.1 (644.7–807.3)	0.508	−0.1 (−79.1–25.7)	0.364	0.070	0.496
F2-isoprostanes (pg/mL)	435.7 (280.0–568.8)	476.4 (417.8–859.6)	0.386	65.2 (−92.7–246.9)	512.8 (335.9–643.6)	639.8 (303.7–858.8)	0.093	82.4 (−10.9–198.9)	0.364	0.762	0.650
TNFSR-II (ng/mL)	0.666 (0.214–2.693)	0.966 6 (0.461–3.721)	0.799	−0.126 (−0.655–0.709)	0.464 (0.348–0.780)	0.662 (0.473–1.225)	0.169	0.220 (−0.097–0.408)	0.597	0.496	0.496
WBC (×10^9^/L)	8.2 (6.1–9.5)	8.6 (7.4–10.5)	**0.037**	0.8 (0.0–2.2)	10.0 (7.1–13.5)	9.1 (7.3–12.4)	0.445	−0.5 (−1.8–1.0)	0.131	0.762	0.070
Neutrophils (%)	70.1 (64.6–72.8)	68.1 (63.7–75.0)	0.959	−0.3 (−4.1–2.9)	68.9 (67.2–77.4)	67.4 (63.8–75.7)	0.092	−2.9 (−4.7–0.7)	0.970	0.821	0.241
Neutrophils (×10^9^/L)	5.7 (4.2–6.6)	5.9 (5.1–7.5)	0.095	0.6 (−0.1–1.2)	6.8 (4.5–9.4)	6.4 (4.7–8.8)	0.285	−0.7 (−1.6–0.7)	0.131	0.597	0.096
Monocytes (%)	4.5 (4.0–5.4)	4.3 (4.0–4.9)	1.000	0.1 (−0.8–0.2)	4.0 (3.3–5.1)	4.2 (3.4–5.0)	0.330	0.2 (−0.3–0.7)	0.345	0.705	0.425
Monocytes (×10^9^/L)	0.32 (0.26–0.50)	0.37 (0.28–0.50)	0.241	0.06 (−0.03–0.13)	0.37 (0.28–0.48)	0.42 (0.31–0.47)	0.799	−0.03 (−0.09–0.11)	0.705	0.880	0.326
NLR	3.2 (2.4–3.9)	3.1 (2.5–4.6)	0.878	0.0 (−0.6–0.5)	3.0 (2.9–5.6)	2.9 (2.3–4.7)	0.139	−0.3 (−1.0–0.1)	1.000	0.821	0.326
Lymphocytes (%)	22.0 (18.8–28.2)	21.4 (16.3–26.7)	0.445	−1.0 (−3.2–2.4)	22.7 (14.1–23.6)	22.9 (16.2–28.1)	0.203	1.2 (−0.6–4.1)	1.000	0.821	0.064
Lymphocytes (×10^9^/L)	2.0 (1.2–2.2)	2.0 (1.4–2.7)	0.093	0.3 (-0.1–0.4)	1.9 (1.5–3.2)	2.2 (1.6–3.1)	0.508	0.0 (-0.1–0.3)	0.406	0.545	0.364
T lymphocytes CD3+ (%)	71.0 (67.1–77.9)	69.1 (53.1–74.4)	0.508	–0.5 (−13.9–3.4)	57.8 (49.2–67.9)	59.4 (45.7–68.5)	0.959	−0.5 (−5.9–6.1)	0.059	0.290	0.597
T lymphocytes CD3+ (×10^9^/L)	1.4 (0.7–1.8)	1.3 (0.9–1.9)	0.241	0.1 (−0.1–0.3)	1.2 (0.7–1.7)	1.4 (0.6–1.7)	0.445	0.1 (−0.2–0.3)	0.705	0.940	0.705
T helper cells CD3+CD4+ (%)	42.0 (28.4–48.5)	40.3 (30.0–45.6)	0.759	−2.0 (−2.5–2.9)	41.5 (35.0–51.2)	43.8 (34.5–49.8)	0.445	−0.2 (−2.7–1.2)	0.880	0.545	0.940
T helper cells CD3+CD4+ (×10^9^/L)	0.41 (0.33–0.63)	0.45 (0.36–0.55)	0.203	0.05 (−0.01–0.08)	0.44 (0.25–0.79)	0.61 (0.18–0.77)	0.646	−0.01 (−0.07–0.08)	1.000	0.496	0.496
Activated T helper cells CD3+CD4+CD25+ (%)	35.8 (21.4–46.1)	30.6 (22.2–44.9)	0.515	−0.8 (−2.5–1.7)	25.7 (16.1–41.1)	23.7 (19.1–35.0)	0.878	2.1 (−5.6–3.7)	0.307	0.290	0.677
Activated T helper cells CD3+CD4+CD25+ (×10^9^/L)	0.16 (0.10–0.22)	0.14 (0.09–0.21)	0.575	0.01 (−0.02–0.03)	0.13 (0.07–0.19)	0.13 (0.05–0.21)	0.959	−0.00 (−0.03–0.03)	0.545	0.650	0.821
Cytotoxic T lymphocytes CD3+CD8+ (%)	44.2 (33.9–56.7)	44.5 (36.9–58.7)	0.285	2.2 (−2.0–4.6)	36.7 (27.8–44.0)	34.2 (30.3–41.7)	0.721	0.9 (−2.1–2.6)	0.290	0.131	0.408
Cytotoxic T lymphocytes CD3+CD8+ (×10^9^/L)	0.57 (0.26–0.89)	0.57 (0.35–1.02)	0.093	0.07 (−0.02–0.13)	0.39 (0.24–0.64)	0.46 (0.21–0.70)	0.386	0.04 (−0.08–0.12)	0.290	0.406	0.650
Activated cytotoxic T lymphocytes CD3+CD8+CD25+ (%)	8.8 (5.7–15.4)	7.4 (4.3–12.4)	0.241	−1.3 (−2.8–0.9)	9.5 (3.8–14.1)	7.8 (3.1–16.7)	0.646	0.0 (−5.1–1.5)	0.821	0.940	0.733
Activated cytotoxic T lymphocytes CD3+CD8+CD25+ (×10^9^/L)	0.05 (0.03–0.08)	0.04 (0.03–0.06)	0.878	−0.00 (−0.01–0.01)	0.04 (0.01–0.07)	0.04 (0.02–0.06)	0.646	−0.00 (−0.03–0.01)	0.496	0.762	0.940
T helper cells/cytotoxic T lymphocytes ratio (CD4/CD8)	1.0 (0.5–1.4)	0.9 (0.5–1.2)	0.508	−0.0 (−0.1–0.1)	1.1 (0.9–1.6)	1.3 (0.9–1.5)	0.508	−0.0 (−0.2–0.1)	0.450	0.257	1.000
DPT CD3+CD4+CD8+ (%)	0.6 (0.5–1.7)	0.7 (0.5–1.3)	0.766	0.1 (−0.3–0.2)	1.0 (0.6–2.1)	1.0 (0.7–2.7)	0.528	0.1 (−0.3–0.5)	0.206	0.266	0.733
DPT CD3+CD4+CD8+ (×10^9^/L)	0.008 (0.007–0.016)	0.010 (0.007–0.019)	0.386	0.000 (−0.001–0.005)	0.013 (0.009–0.018)	0.015 (0.007–0.027)	0.386	0.001 (−0.002–0.012)	0.326	0.257	0.762
DNT CD3+CD4-CD8- (%)	15.2 (11.9–17.0)	14.6 (10.6–16.2)	0.285	−1.5 (−4.0–0.8)	16.5 (10.8–20.4)	18.2 (10.4–20.8)	0.953	−0.1 (−1.0–1.3)	0.820	0.450	0.199
DNT CD3+CD4-CD8- (×10^9^/L)	0.17 (0.11–0.27)	0.16 (0.13–0.23)	0.799	0.02 (−0.07–0.02)	0.15 (0.10–0.24)	0.16 (0.09–0.27)	0.878	−0.01 (−0.03–0.05)	1.000	0.940	0.880
B lymphocytes CD45+CD21+ (%)	12.3 (8.3–17.2)	13.9 (7.4–16.7)	0.919	−0.1 (−1.4–2.0)	16.9 (10.2–24.6)	13.5 (10.1–22.0)	0.110	−1.5 (−2.3–0.2)	0.130	0.427	0.089
B lymphocytes CD45+CD21+ (×10^9^/L)	0.19 (0.13–0.32)	0.22 (0.14–0.32)	0.285	0.03 (−0.03–0.06)	0.32 (0.13–0.78)	0.29 (0.13–0.68)	0.575	-0.00 (−0.10–0.03)	0.290	0.364	0.364
NT-proBNP (pmol/L)	646.5 (440.3–1031.3)	712.0 (455.0–1044.0)	0.541	0.0 (−143.0–236.3)	974.0 (867.0–1299.0)	953.0 (602.5–1247.5)	0.515	3.0 (−238.0–49.5)	0.102	0.288	0.450
cTnI (µg/L)	0.048 (0.037–0.099)	0.044 (0.031–0.067)	0.959	0.003 (−0.016–0.011)	0.043 (0.030–0.075)	0.039 (0.025–0.066)	0.678	−0.004 (−0.010–0.008)	0.450	0.762	0.970
LA/Ao	2.0 (1.8–2.0)	1.8 (1.5–2.0)	0.333	−0.1 (−0.4–0.1)	1.9 (1.8–2.2)	2.0 (1.7–2.3)	0.721	−0.0 (−0.2–0.2)	0.970	0.272	0.650
nLVIDd	1.7 (1.6–1.8)	1.7 (1.6–1.8)	0.878	−0.0 (−0.1–0.2)	1.9 (1.8–2.0)	1.9 (1.9–2.0)	0.878	0.0 (−0.1–0.1)	**0.034**	0.082	1.000
nLVIDs	0.9 (0.8–1.0)	1.0 (0.8–1.0)	0.333	0.0 (−0.1–0.2)	1.0 (0.8–1.2)	1.0 (0.8–1.1)	0.878	0.0 (−0.1–0.1)	0.290	0.597	0.290
MV E velocity (m/s)	0.96 (0.88–1.09)	0.90 (0.82–1.00)	0.161	−0.10 (-0.21–0.05)	0.87 (0.84–1.14)	1.04 (0.80–1.20)	0.475	0.01 (−0.09–0.17)	0.657	0.328	0.197
MV A velocity (m/s)	0.84 (0.59–0.90)	0.75 (0.65–0.91)	0.889	0.01 (−0.13–0.12)	0.75 (0.60–0.98)	0.74 (0.59–0.82)	0.507	−0.03 (−0.12–0.10)	0.965	0.756	0.503
MV E/A	1.18 (1.05–1.61)	1.08 (1.00–1.30)	0.575	−0.04 (−0.40–0.27)	1.15 (0.96–1.56)	1.25 (1.05–1.69)	0.114	0.17 (−0.01–0.27)	0.477	0.286	0.286
TR PG (mmHg)	28.0 (20.0–33.0)	29.0 (26.0–51.0)	0.259	6.0 (−6.0–16.5)	28.0 (25.0–50.0)	33.0 (27.0–34.0)	1.000	3.0 (−13.0–7.9)	0.424	0.915	0.367
FS (%)	47.5 (41.8–49.3)	45.0 (38.0–51.0)	0.261	−2.0 (−8.5–2.3)	44.0 (40.5–55.3)	44.0 (41.0–53.5)	0.799	0.0 (−2.3–1.5)	0.791	0.363	0.448
HR (bpm)	130.0 (119.0–150.0)	124.0 (100.0–142.5)	0.321	−2.0 (−12.5–8.5)	135.0 (117.5–140.0)	125.0 (110.0–142.5)	0.713	−10.0 (−10.0–12.5)	0.818	0.676	0.907
Murmur (grade)	4.0 (3.8–4.0)	4.0 (3.0–4.0)	0.157	0.0 (−0.3–0.0)	4.0 (4.0–4.0)	4.0 (4.0–4.0)	0.317	0.0 (0.0–0.0)	0.542	0.194	0.542
Weight (kg)	7.7 (6.0–10.7)	8.2 (5.7–11.2)	0.799	0.0 (−0.2–0.3)	9.2 (7.2–13.8)	9.1 (7.1–14.3)	0.721	−0.0 (−0.3–0.2)	0.257	0.384	0.791
BCS	5.0 (5.0–6.3)	5.0 (5.0–6.3)	1.000	0.0 (0.0–0.0)	5.0 (4.8–5.3)	5.0 (4.0–6.0)	0.655	0.00 (0.00–0.00)	0.170	0.319	0.957

* p-values resulting from the comparison (Wilcoxon test) of parameters between baseline and the end of the supplementation period in the same group; ^a^ *p*-values resulting from the comparison (Mann–Whitney test) of parameters between the placebo and the CoQ_10_ groups at baseline; ^b^ *p*-values resulting from the comparison (Mann–Whitney test) of parameters between the placebo and the CoQ_10_ groups after a 3-month supplementation period; ^c^ *p*-values resulting from the comparison (Mann–Whitney test) of delta values between the placebo and CoQ_10_ groups. Significant results (*p* < 0.05) are in bold. Abbreviations: A, A-wave (late transmitral blood flow); ACVIM, American College of Veterinary Internal Medicine; BCS, body condition score; cardiac TnI, cardiac troponin I; CD, cluster of differentiation; CoQ_10_, coenzyme Q_10_; DNT, double-negative T lymphocytes; DPT, double-positive T lymphocytes; E, E-wave (early transmitral blood flow); FS, fractional shortening; GPX, glutathione peroxidase; HR, heartrate; IQR, interquartile range; LA/Ao, left atrium to aorta ratio; MMVD, myxomatous mitral valve disease; MV, mitral valve; NLR, neutrophil-to-lymphocyte ratio; nLVIDd, normalized end-diastole left ventricular internal dimension; nLVIDs, normalized end-systole left ventricular internal dimension; NT-proBNP, N-terminal pro B-type natriuretic peptide; TNFα, tumor necrosis factor α; TNFSR-II, tumor necrosis factor soluble receptor II; TR PG, tricuspid regurgitation peak gradient; WBC, white blood cells.

**Table 3:** Effect of CoQ_10_ (or placebo) supplementation on selected parameters in dogs with MMVD and CHF. Medians and interquartile ranges (IQR) are shown.

Parameter		CoQ_10_ Supplemented			Placebo						
	Baseline	After 3 Months	*p* *	Delta	Baseline	After 3 Months	*p* *	Delta	*p* ^a^	*p* ^b^	*p* ^c^
CoQ_10_ (mg/L)	0.171 (0.144–0.222)	0.863 (0.598–1.247)	**0.001**	0.705 (0.371–1.071)	0.164 (0.142–0.230)	0.163 (0.138–0.208)	0.575	0.007 (−0.033–0.034)	0.804	**<0.001**	**<0.001**
GPX (U/g HGB)	807.3 (690.8–823.9)	765.9 (711.1–812.3)	0.701	−14.8 (−41.6–32.1)	786.4 (723.3–854.3)	827.8 (729.2–894.1)	0.139	30.8 (7.7–57.7)	0.664	0.137	0.137
F2-isoprostanes (pg/mL)	510.6 (405.7–680.8)	534.1 (467.7–649.8)	0.917	7.4 (−104.2–90.9)	468.2 (338.1–737.8)	658.5 (399.1–813.3)	0.169	85.7 (−35.7–169.5)	0.804	0.756	0.239
TNFSR-II (ng/mL)	2.144 (1.292–3.278)	1.113 (0.802–1.683)	0.071	−0.650 (−2.059–0.270)	0.983 (0.607–4.312)	1.149 (0.713–2.947)	0.515	−0.007 (−1.559–0.322)	0.598	0.843	0.391
WBC (×10^9^/L)	8.8 (6.5–10.0)	9.8 (7.2–11.3)	0.249	0.8 (−0.7–1.3)	9.9 (7.1–12.7)	9.3 (8.0–13.1)	0.799	−0.6 (−1.2–1.4)	0.385	0.577	0.420
Neutrophils (%)	69.3 (63.9–73.9)	67.4 (64.7–72.5)	**0.363**	−2.2 (−4.6–3.0)	66.4 (58.1–71.4)	68.6 (62.7–76.2)	0.241	4.2 (−1.1–10.3)	0.336	0.852	**0.041**
Neutrophils (×10^9^/L)	5.9 (4.4–7.6)	6.0 (4.9–8.1)	0.345	0.5 (−0.6–0.9)	5.9 (4.8–8.6)	6.9 (5.5–7.8)	0.721	0.0 (−1.0–1.4)	0.756	0.577	0.901
Monocytes (%)	5.1 (4.3–6.2)	5.2 (4.1–6.4)	0.527	−0.2 (−0.5–0.7)	5.1 (4.0–6.3)	4.8 (3.1–5.4)	0.386	−0.6 (−1.5–0.6)	0.664	0.351	0.192
Monocytes (×10^9^/L)	0.46 (0.36–0.61)	0.47 (0.40–0.59)	0.311	0.01 (−0.04–0.09)	0.49 (0.35–0.66)	0.45 (0.37–0.56)	0.445	−0.01 (−0.21–0.13)	0.535	0.664	0.121
NLR	3.3 (2.5–4.6)	3.2 (2.6–3.8)	0.345	−0.3 (−0.8–0.5)	3.0 (2.3–3.8)	3.7 (2.5–4.9)	0.093	0.8 (0.3–1.2)	0.620	0.577	0.055
Lymphocytes (%)	21.5 (16.2–24.4)	22.3 (18.6–25.3)	0.311	1.3 (−2.5–3.9)	22.1 (17.7–28.3)	18.7 (15.7–25.8)	0.139	−3.5 (−9.0– −0.3)	0.598	0.438	**0.044**
Lymphocytes (×10^9^/L)	1.6 (1.4–2.1)	2.0 (1.4–2.6)	0.152	0.0 (−0.2–0.5)	2.0 (1.6–3.2)	1.9 (1.1–3.1)	0.203	−0.3 (−0.7–0.1)	0.107	0.804	**0.041**
T lymphocytes CD3+ (%)	61.6 (50.3–70.7)	63.2 (42.4–74.5)	0.600	1.5 (−5.3–6.6)	58.6 (22.9–64.9)	58.7 (33.5–66.1)	0.169	5.4 (−4.5–15.0)	0.264	0.420	0.264
T lymphocytes CD3+ (×10^9^/L)	1.0 (0.8–1.3)	1.0 (0.8–1.5)	0.152	0.0 (−0.1–0.3)	0.9 (0.4–1.9)	1.2 (0.5–1.8)	0.878	−0.0 (−0.1–0.1)	0.951	0.951	0.352
T helper cells CD3+CD4+ (%)	50.5 (46.5–60.0)	53.1 (45.5–54.7)	0.093	−2.8 (−6.4–1.1)	52.3 (41.7–54.5)	49.3 (44.6–55.7)	0.721	0.5 (−3.6–3.5)	0.385	0.951	0.154
T helper cells CD3+CD4+ (×10^9^/L)	0.47 (0.38–0.72)	0.46 (0.37–0.76)	0.861	−0.00 (−0.09–0.08)	0.45 (0.19–1.00)	0.58 (0.28–0.82)	0.646	−0.01 (−0.09–0.09)	0.804	0.852	1.000
Activated T helper cells CD3+CD4+CD25+ (%)	22.6 (19.2–42.3)	22.7 (17.9–30.6)	0.505	−1.5 (−4.6–2.0)	30.5 (26.8–33.3)	27.2 (25.2–32.6)	**0.037**	−3.5 (−5.6– −0.9)	0.131	0.313	0.402
Activated T helper cells CD3+CD4+CD25+ (×10^9^/L)	0.12 (0.08–0.24)	0.11 (0.09–0.17)	0.650	−0.01 (−0.04–0.03)	0.15 (0.05–0.25)	0.15 (0.09–0.20)	0.445	−0.01 (−0.09–0.03)	1.000	0.535	0.951
Cytotoxic T lymphocytes CD3+CD8+ (%)	29.1 (23.3–33.3)	32.3 (23.0–39.9)	0.075	2.1 (−0.6–8.4)	28.1 (23.8–34.2)	30.3 (25.7–35.9)	0.343	1.3 (−1.7–4.9)	0.877	0.901	0.515
Cytotoxic T lymphocytes CD3+CD8+ (×10^9^/L)	0.28 (0.21–0.39)	0.37 (0.18–0.54)	0.075	0.01 (−0.01–0.19)	0.27 (0.12–0.65)	0.36 (0.14–0.59)	0.386	0.02 (−0.04–0.06)	0.852	0.756	0.420
Activated cytotoxic T lymphocytes CD3+CD8+CD25+ (%)	9.2 (6.0–14.7)	8.3 (5.7–10.6)	0.239	−0.5 (−3.9–1.4)	12.2 (8.7–16.7)	12.4 (5.7–15.7)	0.168	−1.1 (−1.7–0.8)	0.251	0.264	0.901
Activated cytotoxic T lymphocytes CD3+CD8+CD25+ (×10^9^/L)	0.03 (0.02–0.04)	0.03 (0.02–0.04)	0.917	−0.00 (−0.01–0.01)	0.03 (0.01–0.07)	0.03 (0.02–0.06)	0.799	−0.00 (−0.01–0.01)	1.000	0.951	0.710
T helper cells/cytotoxic T lymphocytes ratio (CD4/CD8)	1.6 (1.4–2.6)	1.6 (1.2–2.5)	0.133	–0.3 (–0.5–0.1)	1.9 (1.3–2.1)	1.6 (1.4–2.0)	0.445	−0.0 (−0.5–0.2)	0.710	0.804	0.535
DPT CD3+CD4+CD8+ (%)	1.2 (0.8–2.1)	1.0 (0.8–1.9)	0.592	−0.1 (−0.3–0.2)	1.2 (0.8–2.0)	1.2 (1.0–2.2)	0.252	0.1 (−0.1–0.3)	0.975	0.454	0.235
DPT CD3+CD4+CD8+ (×10^9^/L)	0.011 (0.005–0.020)	0.012 (0.005–0.024)	0.972	−0.001 (−0.002–0.004)	0.012 (0.007–0.018)	0.014 (0.007–0.022)	0.575	0.000 (−0.002–0.005)	0.901	0.951	0.852
DNT CD3+CD4-CD8- (%)	17.1 (12.9–24.3)	17.2 (11.2–22.7)	**0.046**	−1.7 (−2.6–-0.2)	19.5 (15.0–20.9)	16.9 (13.0–19.4)	**0.028**	−2.1 (−3.0– −0.2)	0.828	0.975	0.576
DNT CD3+CD4-CD8- (×10^9^/L)	0.17 (0.12–0.22)	0.17 (0.11–0.22)	0.972	-0.00 (−0.03–0.03)	0.18 (0.09–0.34)	0.19 (0.10–0.27)	0.241	−0.01 (−0.07–0.01)	0.901	0.804	0.385
B lymphocytes CD45+CD21+ (%)	14.8 (11.8–26.0)	18.5 (13.0–23.2)	0.221	1.0 (−2.0–5.4)	17.3 (15.8–20.1)	14.2 (12.8–21.1)	0.285	−3.3 (−4.6–2.5)	0.535	0.336	0.107
B lymphocytes CD45+CD21+ (×10^9^/L)	0.31 (0.18–0.40)	0.34 (0.25–0.53)	0.152	0.02 (−0.03–0.25)	0.39 (0.27–0.58)	0.26 (0.15–0.67)	0.333	−0.04 (−0.14–0.06)	0.215	0.664	0.121
NT-proBNP (pmol/L)	1600.0 (647.5–2356.5)	1344.0 (767.5–4472.0)	0.382	117.0 (−177.5–1798.0)	2688.0 (1730.0–4271.5)	2647.0 (1215.5–4917.3)	0.799	−35.0 (−867.0–1848.0)	0.137	0.385	0.577
cTnI (µg/L)	0.060 (0.030–0.090)	0.064 (0.039–0.166)	0.249	0.013 (−0.010–0.035)	0.090 (0.062–0.173)	0.135 (0.051–0.263)	0.326	0.004 (−0.023–0.076)	0.117	0.216	0.841
LA/Ao	2.2 (2.1–2.4)	2.2 (2.0–2.4)	0.646	0.0 (−0.2–0.2)	2.2 (2.0–2.3)	2.0 (1.8–2.5)	0.760	−0.1 (−0.2–0.4)	0.344	0.256	0.940
nLVIDd	2.0 (2.0–2.2)	2.1 (1.9–2.3)	0.695	0.0 (−0.1–0.2)	2.1 (1.7–2.4)	1.8 (1.6–2.4)	0.333	−0.1 (−0.4–0.1)	0.895	0.391	0.391
nLVIDs	1.0 (0.7–1.2)	1.1 (0.9–1.4)	0.060	0.2 (0.0–0.3)	1.0 (0.8–1.5)	0.8 (0.7–1.3)	**0.022**	−0.1 (−0.2– −0.0)	0.553	0.391	**0.006**
MV E velocity (m/s)	1.29 (1.12–1.51)	1.18 (1.01–1.68)	0.638	0.05 (−0.14–0.18)	1.19 (1.03–1.53)	1.15 (0.81–1.40)	0.074	−0.13 (−0.22–0.06)	0.368	0.420	0.162
MV A velocity (m/s)	0.79 (0.72–1.02)	0.84 (0.65–0.97)	0.328	-0.04 (−0.15–0.10)	0.85 (0.79–0.96)	0.90 (0.60–1.13)	0.646	−0.05 (−0.15–0.13)	0.620	0.828	0.780
MV E/A	1.49 (1.14–1.85)	1.57 (1.21–1.73)	0.972	0.06 (−0.43–0.33)	1.49 (1.18–1.61)	1.29 (1.06–1.71)	0.285	−0.04 (−0.29–0.12)	0.710	0.239	0.756
TR PG (mmHg)	40.0 (35.8–51.8)	41.0 (35.5–50.0)	0.859	−2.0 (−12.3–10.0)	38.0 (36.5–53.5)	34.0 (26.5–48.5)	0.373	−5.9 (−14.0–7.5)	0.902	0.306	0.567
FS (%)	47.5 (44.0–61.8)	47.0 (36.0–48.8)	**0.049**	−5.5 (−10.3–-4)	44.7 (33.8–52.0)	45.5 (38.3–58.5)	0.105	2.5 (−0.3–8.8)	0.321	0.843	**0.007**
HR (bpm)	140.0 (110.0–145.0)	130.0 (120.0–150.0)	0.632	10.0 (−15.0–10.0)	133.0 (120.0–142.5)	130.0 (110.0–142.5)	0.671	−5.0 (−16.5–20.0)	0.850	0.684	0.490
Murmur (grade)	4.00 (4.0–5.0)	4.00 (4.0–4.5)	0.083	0.0 (−0.5–0.0)	4.0 (4.0–5.0)	4.5 (4.0–5.0)	0.564	0.0 (0.0–0.3)	0.442	0.365	0.136
Weight (kg)	8.0 (5.2–11.0)	8.0 (4.7–10.5)	0.328	−0.2 (−0.6–0.1)	7.8 (6.9–11.6)	8.1 (7.1–11.6)	0.285	0.2 (−0.3–0.6)	0.620	0.535	0.163
BCS	5.0 (4.5–6.5)	5.0 (3.5–6.5)	0.206	0.0 (−1.0–0.0)	4.0 (3.75–6.0)	4.5 (3.75–6.25)	0.083	0.0 (0.0–1.0)	0.127	0.775	0.062

* p-values resulting from the comparison (Wilcoxon test) of parameters between baseline and the end of the supplementation period in the same group; ^a^ *p*-values resulting from the comparison (Mann–Whitney test) of parameters between the placebo and CoQ_10_ groups at baseline; ^b^ *p*-values resulting from the comparison (Mann–Whitney test) of parameters between the placebo and CoQ_10_ groups after a 3-month supplementation period; ^c^ *p*-values resulting from the comparison (Mann–Whitney test) of delta values between the placebo and CoQ_10_ groups. Significant results (*p* < 0.05) are in bold. Abbreviations: A, A-wave (late transmitral blood flow); ACVIM, American College of Veterinary Internal Medicine; BCS, body condition score; cardiac TnI, cardiac troponin I; CD, cluster of differentiation; CoQ_10_, coenzyme Q_10_; DNT, double-negative T lymphocytes; DPT, double-positive T lymphocytes; E, E-wave (early transmitral blood flow); FS, fractional shortening; GPX, glutathione peroxidase; HR, heartrate; IQR, interquartile range; LA/Ao, left atrium to aorta ratio; MMVD, myxomatous mitral valve disease; MV, mitral valve; NLR, neutrophil-to-lymphocyte ratio; nLVIDd, normalized end-diastole left ventricular internal dimension; nLVIDs, normalized end-systole left ventricular internal dimension; NT-proBNP, N-terminal pro B-type natriuretic peptide; TNFα, tumor necrosis factor α; TNFSR-II, tumor necrosis factor soluble receptor II; TR PG, tricuspid regurgitation peak gradient; WBC, white blood cells.

**Table 4:** Baseline measurements in MMVD (ACVIM B2 and CHF) and healthy dogs. Medians and interquartile ranges (IQR) are shown.

Parameter	ACVIM B2	CHF	Healthy	*p* *	*p* ^a^	*p* ^b^	*p* ^c^
CoQ_10_ (mg/L)	0.176 (0.125–0.213)	0.171 (0.145–0.213)	0.095 (0.070–0.143)	**0.006**	**0.019**	**0.008**	1.000
GPX (U/g HGB)	746.5 (675.8–792.1)	792.6 (692.0–827.7)	775.4 (684.1–827.5)	0.317	/	/	/
F2-isoprostanes (pg/mL)	468.9 (323.2–586.1)	510.6 (356.2–727.0)	536.0 (420.2–884.2)	0.373	/	/	/
TNFSR-II (ng/mL)	0.539 (0.247–0.949)	1.716 (0.683–3.426)	0.752 (0.495–0.876)	**0.040**	1.000	**0.048**	**0.006**
WBC (×10^9^/L)	8.4 (6.9–10.7)	9.0 (7.1–10.9)	8.3 (5.9–9.5)	0.463	/	/	/
Neutrophils (%)	69.4 (66.8–74.7)	68.9 (63.3–72.5)	66.9 (62.6–72.9)	0.642	/	/	/
Neutrophils (×10^9^/L)	5.7 (4.9–8.1)	5.9 (4.8–7.8)	5.6 (4.1–6.6)	0.556	/	/	/
Monocytes (%)	4.3 (3.6–5.1)	5.1 (4.1–6.2)	4.1 (3.3–5.7)	0.120	/	/	/
Monocytes (×10^9^/L)	0.35 (0.28–0.46)	0.47 (0.36–0.63)	0.34 (0.27–0.49)	**0.044**	1.000	0.122	0.099
NLR	3.0 (2.9–4.5)	3.2 (2.6–4.3)	2.7 (2.3–4.0)	0.654	/	/	/
Lymphocytes (%)	22.5 (16.9–23.7)	21.5 (16.9–25.4)	24.3 (18.6–27.7)	0.624	/	/	/
Lymphocytes (×10^9^/L)	1.9 (1.3–2.6)	1.8 (1.5–2.5)	1.8 (1.4–2.5)	0.877	/	/	/
T lymphocytes CD3+ (%)	67.1 (53.6–75.9)	61.0 (30.5–67.2)	60.5 (53.9–71.7)	0.180	/	/	/
T lymphocytes CD3+ (×10^9^/L)	1.22 (0.76–1.73)	0.96 (0.62–1.55)	1.04 (0.72–1.66)	0.640	/	/	/
T helper cells CD3+CD4+ (%)	41.7 (31.2–47.9)	52.0 (44.7–56.6)	53.6 (45.2–62.8)	**0.003**	**0.012**	1.000	**0.013**
T helper cells CD3+CD4+ (×10^9^/L)	0.41 (0.31–0.73)	0.47 (0.35–1.55)	0.55 (0.42–0.73)	0.314	/	/	/
Activated T helper cells CD3+CD4+CD25+ (%)	30.2 (19.5–44.7)	26.0 (21.4–32.8)	35.7 (23.0–42.0)	0.497	/	/	/
Activated T helper cells CD3+CD4+CD25+ (×10^9^/L)	0.14 (0.08–0.20)	0.13 (0.07–0.25)	0.19 (0.16–0.24)	0.910	/	/	/
Cytotoxic T lymphocytes CD3+CD8+ (%)	39.1 (31.1–50.1)	29.1 (24.0–33.2)	26.0 (20.3–34.8)	**0.003**	**0.020**	1.000	**0.007**
Cytotoxic T lymphocytes CD3+CD8+ (×10^9^/L)	0.47 (0.26–0.76)	0.28 (0.14–0.40)	0.30 (0.19–0.50)	0.080	/	/	/
Activated cytotoxic T lymphocytes CD3+CD8+CD25+ (%)	9.1 (5.2–14.0)	10.1 (6.2–14.7)	12.5 (10.6–22.6)	0.178	/	/	/
Activated cytotoxic T lymphocytes CD3+CD8+CD25+ (×10^9^/L)	0.05 (0.03–0.07)	0.03 (0.01–0.05)	0.07 (0.03–0.09)	0.456	/	/	/
T helper cells/cytotoxic T lymphocytes ratio (CD4/CD8)	1.05 (0.72–1.40)	1.75 (1.42–2.28)	2.17 (1.31–2.76)	**0.001**	**0.007**	1.000	**0.003**
DPT CD3+CD4+CD8+ (%)	0.7 (0.5–1.8)	1.2 (0.8–2.0)	1.2 (0.9–1.5)	0.295	/	/	/
DPT CD3+CD4+CD8+ (×10^9^/L)	0.011 (0.007–0.015)	0.011 (0.007–0.019)	0.014 (0.008–0.027)	0.719	/	/	/
DNT CD3+CD4-CD8- (%)	15.9 (11.7–18.1)	17.8 (13.5–23.9)	15.8 (12.1–20.5)	0.255	/	/	/
DNT CD3+CD4-CD8- (×10^9^/L)	0.15 (0.11–0.25)	0.17 (0.11–0.26)	0.15 (0.10–0.31)	0.985	/	/	/
B lymphocytes CD45+CD21+ (%)	14.1 (9.9–18.4)	16.9 (12.6–23.1)	13.7 (10.7–16.8)	0.174	/	/	/
B lymphocytes CD45+CD21+ (×10^9^/L)	0.21 (0.14–0.46)	0.32 (0.20–0.49)	0.25 (0.18–0.35)	0.349	/	/	/
NT-proBNP (pmol/L)	950.0 (492.0–1116.0)	1956.0 (1093.0–2924.0)	1452.0 (1110.0–1853.0)	**0.004**	0.341	0.690	**0.001**
Cardiac TnI (µg/L)	0.044 (0.034–0.086)	0.078 (0.037–0.112)	0.022 (0.012–0.189)	0.087	/	/	/
LA/Ao	1.95 (1.79–2.05)	2.21 (1.98–2.32)	1.36 (1.17–1.60)	**<0.001**	**0.002**	**<0.001**	**0.047**
nLVIDd	1.81 (1.64–1.91)	2.04 (1.85–2.18)	1.36 (1.19–1.66)	**<0.001**	**0.031**	**<0.001**	0.050
nLVIDs	0.90 (0.83–1.03)	1.01 (0.83–1.29)	0.80 (0.66–0.92)	0.066	/	/	/
MV E velocity (m/s)	0.92 (0.85–1.12)	1.28 (1.10–1.53)	0.60 (0.54–0.70)	**<0.001**	**0.006**	**<0.001**	**0.015**
MV A velocity (m/s)	0.79 (0.60–0.91)	0.83 (0.74–0.97)	0.46 (0.39–0.62)	**<0.001**	**0.003**	**<0.001**	0.718
MV E/A	1.17 (1.01–1.56)	1.49 (1.22–1.68)	1.31 (1.04–1.50)	0.175	/	/	/
TR PG (mmHg)^d^	28.0 (21.5–43.3)	39.0 (37.0–49.0)	–	/	/	/	**0.008**
FS (%)	45.5 (41.0–50.8)	44.7 (39.3–58.8)	39.5 (37.0–51.5)	0.556	/	/	/
HR (bpm)	130.0 (120.0–140.0)	136.0 (120.0–140.0)	110.0 (100.0–130.0)	**0.011**	**0.036**	**0.011**	1.000
Murmur (grade)^d^	4.0 (4.0–4.0)	4.0 (4.0–5.0)	–	/	/	/	**<0.001**
BCS	5.0 (5.0–6.0)	5.0 (4.0–6.0)	5.0 (5.0–6.00)	0.205	/	/	/

* p-values resulting from comparison (Kruskal–Wallis test) of parameters between groups of dogs (ACVIM B2, CHF, healthy); ^a^ *p*-values resulting from comparison (multiple comparisons with Bonferroni corrections) between ACVIM B2 and healthy dogs; ^b^ *p*-values resulting from comparison (multiple comparisons with Bonferroni corrections) between CHF and healthy dogs; ^c^ *p*-values resulting from comparison (multiple comparisons with Bonferroni corrections) between ACVIM B2 and CHF dogs; ^d^ Mann–Whitney test was used for comparison of these two parameters between ACVIM B2 and CHF dogs. Significant results (*p* < 0.05) are in bold. Abbreviations: A, A-wave (late transmitral blood flow); ACVIM, American College of Veterinary Internal Medicine; BCS, body condition score; cardiac TnI, cardiac troponin I; CD, cluster of differentiation; CHF, congestive heart failure; CoQ_10_, coenzyme Q_10_; DNT, double-negative T lymphocytes; DPT, double-positive T lymphocytes; E, E-wave (early transmitral blood flow); FS, fractional shortening; GPX, glutathione peroxidase; HR, heartrate; IQR, interquartile range; LA/Ao, left atrium to aorta ratio; MMVD, myxomatous mitral valve disease; MV, mitral valve; NLR, neutrophil-to-lymphocyte ratio; nLVIDd, normalized end-diastole left ventricular internal dimension; nLVIDs, normalized end-systole left ventricular internal dimension; NT-proBNP, N-terminal pro B-type natriuretic peptide; TNFα, tumor necrosis factor α; TNFSR-II, tumor necrosis factor soluble receptor II; TR PG, tricuspid regurgitation peak gradient; WBC, white blood cells.

## Data Availability

The data from this work are included in this article and may be obtained from the corresponding author.
